# BBS Posts Time Series Analysis based on Sample Entropy and Deep Neural Networks

**DOI:** 10.3390/e21010057

**Published:** 2019-01-12

**Authors:** Jindong Chen, Yuxuan Du, Linlin Liu, Pinyi Zhang, Wen Zhang

**Affiliations:** 1 School of Economics and Management, Beijing Information Science & Technology University, Beijing 100192, China; 2 Beijing Key Lab of Green Development Decision Based on Big Data, Beijing 100192, China; 3 School of Economics and Management, Beijing University of Technology, Beijing 100124, China; 4 School of Information Engineering, Xi’an University, Xi’an 710065, China

**Keywords:** sample entropy, deep neural networks, BBS posts, time series

## Abstract

The modeling and forecasting of BBS (Bulletin Board System) posts time series is crucial for government agencies, corporations and website operators to monitor public opinion. Accurate prediction of the number of BBS posts will assist government agencies or corporations in making timely decisions and estimating the future number of BBS posts will help website operators to allocate resources to deal with the possible hot events pressure. By combining sample entropy (SampEn) and deep neural networks (DNN), an approach (SampEn-DNN) is proposed for BBS posts time series modeling and forecasting. The main idea of SampEn-DNN is to utilize SampEn to decide the input vectors of DNN with smallest complexity, and DNN to enhance the prediction performance of time series. Selecting Tianya Zatan new posts as the data source, the performances of SampEn-DNN were compared with auto-regressive integrated moving average (ARIMA), seasonal ARIMA, polynomial regression, neural networks, etc. approaches for prediction of the daily number of new posts. From the experimental results, it can be found that the proposed approach SampEn-DNN outperforms the state-of-the-art approaches for BBS posts time series modeling and forecasting.

## 1. Introduction

The Internet has become a major public opinion formation and diffusion platform [1]. In the Web 2.0 era, Bulletin Board System (BBS), Micro-blog, WeChat, etc. are main sources for public information dissemination, and become the core areas of public opinion monitoring [2]. The numbers of posts, blogs or micro-blogs represent the hot-degree and trend of public opinion, so time series analysis of the numbers of posts, blogs or micro-blogs, such as prediction of the number of new posts, blogs or micro-blogs in a time interval ahead, can be considered as an important signal for government agencies, enterprises and website operators to make decisions. Based on time series analysis of the numbers of posts, blogs or micro-blogs, convincing results are obtained for the election result prediction [3], crisis management [4] and stock market forecasting [5]. Therefore, time series analysis of the numbers of posts, blogs or micro-blogs enable them to monitor the tendency of public opinion, and further support them to make rational planning and actions for public opinion management and guidance [6].

For government agencies and enterprises, the prediction of the numbers of posts, blogs or micro-blogs can help them make decision for at least three reasons [7,8]. First, the prediction of the numbers of posts, blogs or micro-blogs provides government agencies and enterprises with a measure of the trend, scope and duration time of public opinion on their related topics. Second, it is useful to estimate the effort involved in public opinion management and guidance. To different trend types of events or topics, different operation methods and effort investments are required. Generally, the effort involved in public opinion management and guidance is proportional to the number of posts, blogs or micro-blogs. Third, based on the numbers of posts, blogs or micro-blogs, the effectiveness of guidance strategies can be evaluated. According to the positive or negative feedback from public opinion, the guidance strategies can be improved immediately. For website operators, the prediction of the numbers of posts, blogs or micro-blogs can help them allocate resource or strategies on hot topics [9]. Without sufficient resource allocation for hot events, it will make their system delay or crash. Conversely, too many resources allocation will increase their operational cost.

Hence, for effective management and guidance of public opinion, the prediction of the numbers of posts, blogs or micro-blogs is a critical issue. Various approaches are proposed to solve this problem and we can divide the approaches into two kinds: diffusion model and time series model. For diffusion model, the classic mathematical models of diffusion are adopted to establish public opinion, such as Logistic distribution [10], epidemic model [11] and Michaelis–Menten model [12]. The information diffusion process of public opinion is modeled through the classic diffusion model. Based on the identified model, the trend, peak and duration at different stages of public opinion are predicted. For time series model, ignoring the diffusion mechanism of public opinion, the diffusion model of public opinion is constructed only based on time series data. Auto-regressive integrated moving average (ARIMA) [13], artificial neural network [14] and support vector machines [15,16] are the frequently used models for time series data. Due to effectiveness of time series model for public opinion prediction, the time series model is applied for the prediction of the numbers of posts, blogs or micro-blogs.

In Chinese online community, BBS serves as an important social media; the extensive interests and contents are distinct characters of the Chinese BBS sites [17]. As an emerging media and electronic information center, the Chinese BBS sites fulfill the requirements of netizens to be informed and exchange opinions [18]. With the appearance of blogs, twitter, WeChat, etc., the influence of BBS is declining, but, due to the Internet regulations in China, the Chinese BBS sites can offer another channel to express information and spread opinions, and are still an important platform in China. Tianya Club (http://bbs.tianya.cn/), one of the most influential Chinese BBS sites, provides BBS, blogs, etc. services for netizen and consists of many sub-boards for different content/topic discussions, such as Tianya Zatan and Baixing Shengyin [19]. Tianya Zatan board is an important board within Tianya Club, the content of which covers the daily news of current society and personal life. Daily new posts published in Tianya Zatan board are nearly 1000, and millions of clicks and replies are created by netizens [20]. The daily new posts data of Tianya Zatan were selected as the data source for public opinion monitoring.

Takens’ [21] embedding theorem and Sauer et al.’s [22] embedology theorem provide a theoretical foundation for nonlinear dynamical system reconstruction based on its generated time series sequence. Consequently, for the modeling and forecasting of BBS posts time series, two main problems are: (1) Determination of the embedding dimension. A time series can be represented in the so-called “phase space” by a set of delay vectors (DVs), and the embedding dimension defines the size of the DVs. (2) Which model is fit for BBS post number prediction. Approximate entropy is an effective model for the embedding dimension analysis of time series; sample entropy (SampEn) is a modification of approximate entropy [23,24]. With multiple layers and more neurons, deep neural networks (DNN) can detect the features of data, and are more effective for time series prediction [25].

Therefore, by combining SampEn and DNN, an approach SampEn-DNN is proposed to predict BBS new post number time series. SampEn-DNN applies sample entropy to measure the predictability of DVs with different dimensions, selects the dimension of DVs with smallest complexity, and feeds the DVs as the input of DNN to improve the predictive performance of time series. However, in some cases, the single-scale sample entropy cannot really reflect the complexity of a time series, thus, to avoid this issue, multi-scale sample entropy is adopted in this paper. The skipping parameter and the dimension of DVs are tuned by multi-scale sample entropy. The predictive performance of SampEn-DNN for Tianya Zatan new posts time series was investigated. For the combination of sample entropy and DNN for time series modeling and forecasting, both the proposed method and the application area are attempted for the first time. To illustrate the improvements of SampEn-DNN, the performances of ARIMA, seasonal ARIMA, polynomial regression and artificial neural network (ANN) on Tianya Zatan new posts time series analysis were compared.

The rest of the paper is organized as follows. Section 2 presents the related methods for time series analysis. The proposed approach SampEn-DNN is shown in Section 3. Section 4 presents the experimental results of SampEn-DNN and the state-of-the-art approaches for time series analysis of Tianya Zatan new posts. Finally, concluding remarks and future work are given in Section 5.

## 2. Methodology

Time series $X_{t}(t=\;1,\;2,\;3,\;\ldots ,N)$ is an ordering set of observations of a variable over successive periods of time. Time series modeling and forecasting has fundamental importance to various practical domains [26]. Stock exchange data, wind speed, global temperature, etc. are typical examples of time series. The natural temporal ordering feature of time series makes time series analysis different from other data analysis problems, in which there is no natural ordering of the observations. The studies of time series data can be divided into two parts: one is to extract and understand the meaningful statistics and other characteristics of the data, and the other is to predict future values based on previously observed values. 

Parametric approaches are frequently used for time series modeling and forecasting [26]. The state-of-the-art parametric approaches include ARIMA model, seasonal ARIMA model, polynomial regression and ANN. The parametric approaches of time series analysis assume that underlying process is stationary. Generally, a time series $X_{t}(t=\;1,\;2,\;3,\;\ldots ,N)$ is stationary if $E(X_{t}^{2})<\infty$, $E(X_{t})=\alpha$, ∀t∈T={1,2,…,N} and $E(X_{t+r}-\alpha )(X_{t}-\alpha )=\gamma_{X}(r)$, ∀t,t+r∈T={1,2,…,N}, and $\gamma_{X}(r)$ is the auto-covariance function. In other words, for a stationary time series, the variation is finite, the expected values at any time points equals the same value α, and the auto-covariance is merely dependent on their time lag *r* and not dependent on time *t* or  t + r. For example, the simplest stationary time series is white noise. For time series modeling and forecasting, the first issue is to know whether a time series is non-stationary or stationary. The common method for stationary test is augmented Dicker–Fuller (ADF) test [27]. The related approaches for time series modeling and forecasting are presented as follows.

### 2.1. ARIMA Model

ARIMA model is a general class of ARMA model with differencing manipulation on time series data, and ARMA model consists of two parts: autoregressive (AR) model and moving average (MA) model. These models are applied for the fitting of time series data, and aim to describe the autocorrelations in time series.

For a time series $X_{t}(t=\;1,\;2,\;3,\;\ldots ,N)$, assume the number of autoregressive terms as *p*, AR model can be abbreviated as AR(*p*), and expressed as

(1) $$x_{t}=\phi_{1}x_{t-1}+\phi_{2}x_{t-2}+\cdots +\phi_{p}x_{t-p}+\omega_{t}$$

where $x_{t}$ is stationary, $\;\phi_{1},\;\phi_{2},\;\ldots ,\;\phi_{p}$ are constants and $\phi_{p}\neq 0$. $\omega_{t}$ is assumed to be Gaussian white noise with variance $\sigma_{\omega }^{2}$ and zero mean.

Assume the moving average order as *q*, so MA model can be abbreviated as MA(*q*) and expressed as

(2) $$x_{t}=\omega_{t}+\theta_{1}\omega_{t-1}+\theta_{2}\omega_{t-2}+\cdots +\theta_{q}\omega_{t-q}$$

where model parameters are $\theta_{1},\;\theta_{2},\;\ldots ,\;\theta_{q}(\theta_{q}\;\neq \;0)$, and *q* lags are in the moving average.

According to Equations (1) and (2), ARMA model with the autoregressive and the moving average order *p* and *q* can be abbreviated as ARMA(*p*, *q*) and expressed as

(3) $$x_{t}=\phi_{1}x_{t-1}+\phi_{2}x_{t-2}+\cdots +\phi_{p}x_{t-p}+\omega_{t}+\theta_{1}\omega_{t-1}+\theta_{2}\omega_{t-2}+\cdots +\theta_{q}\omega_{t-q}$$

If the mean μ of $x_{t}$ is non-zero, then set $\alpha =\mu (1-\phi_{1}-\cdots -\phi_{p})$, and the ARMA model can be rewritten as

(4) $$x_{t}=\alpha +\phi_{1}x_{t-1}+\phi_{2}x_{t-2}+\cdots +\phi_{p}x_{t-p}+\omega_{t}+\theta_{1}\omega_{t-1}+\theta_{2}\omega_{t-2}+\cdots +\theta_{q}\omega_{t-q}$$

ARIMA model contains differencing manipulation, which is used to transfer a non-stationary time series to a stationary time series. If *L* is a differencing operator, $W_{t}=\;\nabla^{d}x_{t}=\;{\left(1-L\right)}^{d}x_{t}$ conforms to the process ARMA(*p*, *q*). ARIMA model can be denoted as ARIMA(*p*, *d*, *q*). The general expression of ARIMA(*p*, *d*, *q*) is given as Equation (5).

(5) $$W_{t}=\alpha +\phi_{1}W_{t-1}+\phi_{2}W_{t-2}+\cdots +\phi_{p}W_{t-p}+\omega_{t}+\theta_{1}\omega_{t-1}+\theta_{2}\omega_{t-2}+\cdots +\theta_{q}\omega_{t-q}$$

where, through difference by order *d*, the original time series $X_{t}(t=\;1,\;2,\;3,\;\ldots ,N)$ is converted from non-stationary into a stationary time series ***W****_t_*.

### 2.2. Seasonal ARIMA Model

For real issues, most time series show seasonal variation. Seasonal time series mean that there is a similar trend of the observations during the same period (e.g., daily, monthly or yearly) of the time series. Additionally, the observations during the successive periods may also exhibit another seasonal trend.

To address the seasonality and potential seasonal unit root, an extensional ARIMA model called Seasonal ARIMA model is proposed [27]. Assume the periodicity of time series is *s*, Seasonal ARIMA model is given by

(6) $$(1-L^{s}{)}^{D_{s}}W_{t}=\alpha +\phi_{1}(1-L^{s}{)}^{D_{s}}W_{t-1}+\cdots +\phi_{p}(1-L^{s}{)}^{D_{s}}W_{t-p}+\omega_{t}+\theta_{1}\omega_{t-1}+\cdots +\theta_{q}\omega_{t-q}$$

where $(1-L^{s}{)}^{D_{s}}$ is the seasonal differencing operator, and accounts for non-stationarity in observations made in the same period in successive period, $D_{s}=0\;\mathrm{or}\;1$ for s=0 or >1.

### 2.3. Polynomial Regression

For a given dataset $\left(x_{i},y_{i}\right),\;\;i=1,2,\cdots ,N$, *x* is the independent variable and *y* is the dependent variable. Polynomial regression is a form of linear regression to model the relationship between *x* and *y* as an *n*th order polynomial. In general, a polynomial regression fits data to a model of the following form,

(7) $$y_{i}=a_{0}+a_{1}x_{i}+a_{2}x_{i}^{2}+\cdots +a_{n}x_{i}^{n}+\varepsilon_{i}$$

Parameters $a_{1},a_{2},\cdots ,a_{n}$ of polynomial regression are identified by the method of least squares. According to Taylor’s theorem [28], a polynomial regression is the expansion of Taylor series, so it can be used to approximate continuous functions for curve fitting and trend analysis.

### 2.4. Artificial Neural Networks

ANN is a framework for machine learning inspired by biological neural networks. One of the most widely applied models is back propagation neural network (BPNN) [29]. BPNN is a kind of feed-forward network, the connection weights of which are trained by error back propagation algorithm. For a given dataset $\left(x_{i},y_{i}\right),\;\;i=1,2,\cdots ,N$, the training of BPNN includes two parts: one is forward propagation, and the other is back propagation. Forward propagation: The input sample $x_{i}$ is propagated from the input layer, via the hidden layer, to the output layer. The connection weights of BPNN in forward propagation process are maintained constant. Back propagation: The difference (error) between the real value $y_{i}$ and expected output ${\hat{y}}_{i}$ of BPNN is propagated from the output layer to the input layer. The connection weights of BPNN are updated by the error feedback during the process. The objective of BPNN training is to find a set of network weights that minimize the difference between the real value $y_{i}$ and the expect output ${\hat{y}}_{i}$.

## 3. SampEn-DNN

By combining of sample entropy (SampEn) and deep neural networks (DNN), a novel time series modeling and forecasting method SampEn-DNN is proposed to predict the daily number of BBS new posts.

### 3.1. Sample Entropy

For a time series $X_{t}(t=\;1,\;2,\;3,\;\ldots ,N)$, assume its constant time interval as τ. The constant time interval of BBS new posts time series in this study is one day. The SampEn of time series $X_{t}$ can be computed as follows.

First, define the dimension of embedding vector as *m* and tolerance as *r*, such that embedding vector is given as $X_{m}(i)=\left\{x_{i},x_{i+1},\cdots ,\;x_{i+m-1}\right\}$.

Second, the Chebyshev distance $d\left[X_{m}(i),\;X_{m}(j)\right]$(i ≠ j) is used as the distance function [30],

(8) $$d\left[X_{m}(i),\;X_{m}(j)\right]=\underset{k=0,\cdots ,m-1}{\max}(\left|x(i+k)-x(j+k)\right|)$$

Third, the number of $X_{m}(j)$ (1≤j≤N−m,j≠i) that do not exceed the tolerance *r* ($d\left[X_{m}(i),\;X_{m}(j)\right]<r$) is counted and denoted as $n_{i}(m)$, and then the proportion $c_{i}(m)=\frac{n_{i}(m)}{N-m}$ that any $X_{m}(j)$ is close to $X_{m}(i)$ is computed.

Fourth, by averaging over all possible $X_{m}(i)$, the proportion $c(m)=\frac{1}{N-m+1}\sum_{i=1}^{N-m+1}c_{i}(m)$ is estimated.

Fifth, the Chebyshev distance and c(m+1) for embedding vector dimension as *m* + 1 are computed in a similar way.

Sixth, SampEn of $X_{t}(t=\;1,\;2,\;3,\;\ldots ,N)$ is defined as

(9) $$SampEn\left(m,r\right)=-\log\frac{c(m+1)}{c(m)}$$

From the definition, it can be found that *c*(*m* + 1) is not bigger than *c*(*m*), so the *SampEn*(*m, r*) value will be either zero or positive. For time series dataset, a smaller value of *SampEn*(*m, r*) means more self-similarity (predictability) or less noise. To overcome the shortcuts of single-scale SampEn in some special case, multi-scale SampEn is adopted. In multi-scale SampEn, a certain interval between its every element is defined for input vector specified by the skipping parameter δ. Hence, input vector is modified as $X_{m,\;\delta }\left(i\right)=\left\{x_{i},x_{i\;+\;\delta },\cdots ,\;x_{i\;+\;\left(m-\;1\right)\delta }\right\}$, $c{\left(m\right)}_{\delta }$ is expressed as $c{\left(m\right)}_{\delta }=d\left[X_{m,\delta }\left(i\right),\;X_{m,\delta }\left(j\right)\right]<r$, and then SampEn can be given as $SampEn\left(m,\;r,\;\delta \right)=-\;log\left(c{\left(m\;+\;1\right)}_{\delta }/c\left(m_{\delta }\right)\right)$. In this study, the value of tolerance *r* is set as 0.02*std*, where the notation *std* stands for the standard deviation of time series $X_{t}(t=\;1,\;2,\;3,\;\ldots ,N)$ [24].

**Figure 1:**
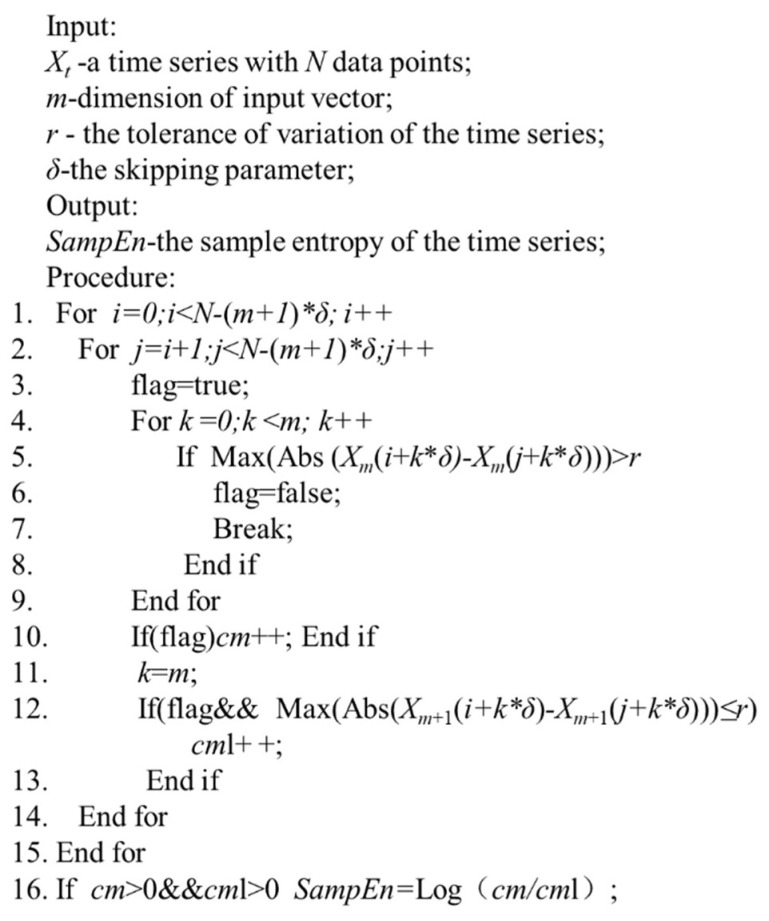
Procedures for the calculation of *SampEn*(*m, r, δ*).

The procedures for the calculation of *SampEn*(*m*, *r*, *δ*) is presented in Figure 1. For the given starting positions as *i* and *j* in the time series $X_{t}(t=\;1,\;2,\;3,\;\ldots ,N)$, Lines 4–9 in Figure 1 are applied to decide $d\left[X_{m,\delta }(i),\;X_{m,\delta }(j)\right]<r$ or not, and Lines 11–13 are implemented to decide $d\left[X_{m+1,\delta }\left(i\right),\;X_{m+1,\delta }\left(j\right)\right]<r$ or not. In Figure 1, for given *i* and *j*, according to the definition of Chebyshev distance, if $d\left[X_{m,\delta }\left(i\right),\;X_{m,\delta }\left(j\right)\right]<r$ and Max(Abs(X(i+k∗δ) −X(j+k∗δ))) ≤r, $d\left[X_{m+1,\delta }(i),\;X_{m+1,\delta }(j)\right]<r$ can be achieved.

### 3.2. Deep Neural Network

**Figure 2:**
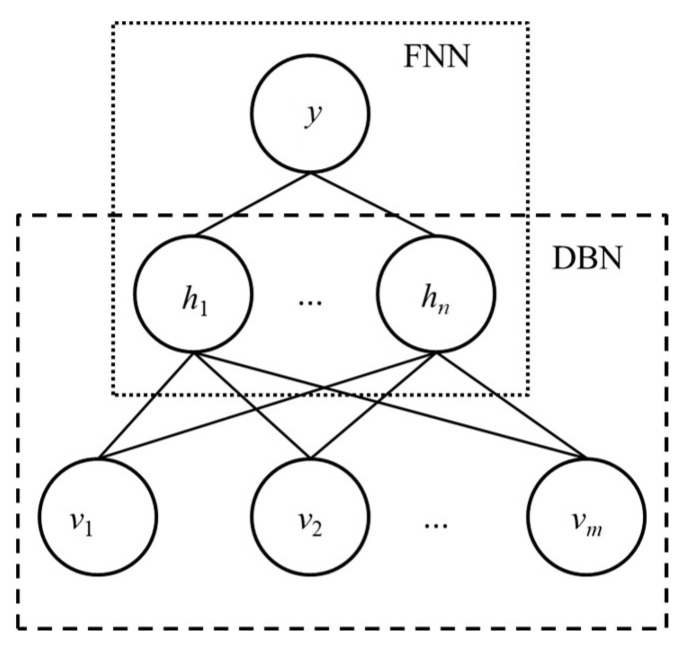
DNN (deep neural networks) model consists of DBN (deep belief network) and FNN (feedforward neural network).

DNN model consists of deep belief network (DBN) [31] and feedforward neural network (FNN). DBN is developed by stacking of multiple-units of Restricted Boltzmann Machines (RBM) [32]. The structure of DNN is shown in Figure 2. The aim of DBN is to extract the high-level features from input data by the stacked RBMs. The learning process of the stacked RBMs is that the features produced by the hidden layer of one RBM serve as the input to the higher-level RBM. The high-level feature representation learned by DBN is fed as the input of FNN. Meanwhile, BPNN is one of the most used FNN models, and adopted for DNN model.

Hence, RBM is the main component of DNN. RBM is an energy-based deep learning model for unsupervised learning, and consists of two kinds of layers: one is the visible layer and the other is the hidden layer. The visible layer is for input data representation, and the hidden layer is to represent a probability of the distribution of input data. The neurons in the visible layer are only connected to the neurons in the hidden layer.

For RBM, assume the numbers of neurons in the visible layer and the hidden layer as *m* and *n*, denoted as $v=(v_{1},\;\ldots ,v_{m})$ and $h=(h_{1},\;\ldots ,h_{n})$, respectively. Meanwhile, assume the *bias* vectors and the weight matrix of RBM as **a**, **b** and **W**. The energy-based model means an entropy function is applied to define the log-likelihood input data distribution over the parameters **a**, **b**, **W**, **v** and **h**. The energy function for RBM is given by Equations (10) and (11).

(10) $$E(v,h)=-h^{T}Wv-a^{T}v-b^{T}h$$

(11) $$E(v,h)=-\sum_{i,j=1}^{m,n}v_{i}h_{j}w_{i,j}-\sum_{i=1}^{m}a_{i}v_{i}-\sum_{j=1}^{n}b_{j}h_{j}$$

For each pair of neurons in the visible layer and the hidden layer, the joint probabilistic distribution is defined as

(12) $$p(v,h)=\frac{e^{-E(v,h)}}{\sum_{v,h}e^{-E(v,h)}}$$

The sum of all probabilities of the hidden vector is the probability that the network assigns to the visible vector, and expressed as

(13) $$p(v)=\frac{\sum_{h}e^{-E(v,h)}}{\sum_{v,h}e^{-E(v,h)}}$$

Since the neurons in visible layer only connect to the neurons in hidden layer, there is no connection between neurons in the same layer. The joint probability of each pair of neurons in different layers can be facilitated by the conditional probabilities,

(14) $$p(h|v)=\prod_{j}p(h_{j}|v)$$

(15) $$p(v|h)=\prod_{i}p(v_{i}|h)$$

For binary data, Equations (14) and (15) can be expressed as

(16) $$p(h_{j}=1|v)=sigm(b_{j}+\sum_{i=1}^{m}v_{i}w_{ij})$$

(17) $$p(v_{i}=1|h)=sigm(a_{i}+\sum_{i=1}^{n}h_{i}w_{ij})$$

where *sigm*(*x*) is the sigmoid function.

### 3.3. SampEn-DNN Approach

DNN as regression model for time series analysis. The primary problem is to decide the formation of its input vectors, which is the main factor to affect the predictive performance for model. Generally, the input vectors are decided by two parameters: the dimension of input vector *m* and the skipping parameter *δ.* Based on SampEn method, the dimension of input vector *m* and the skipping parameter *δ* are optimized. For DNN model training, the first *m* − 1 elements are applied to predict the last (*m-*th) element.

To optimize the parameters *m* and *δ* of input vector, the maximum values of the two parameters need to be decided first. For the dimension of input vector, if *m* is larger than 13, the SampEn of time series cannot be derived in most cases. Because, at this stage, *c(m)* and *c(m +* 1) in Equation (9) are zeros, the differences between the elements of *m*-length and the (*m* + 1)-length input vectors are all bigger than 0.02*std*. For skipping parameter, if the value of *δ* is too big, the intervals between data points will be large, which means the similarity of the data points to each other of time series will be smaller, and the unpredictability of the input vectors will be larger. Based on this point, the biggest skipping parameter *δ* is constrained as 12 in the study [16].

The SampEn results of BBS new posts time series based on different dimensions of input vectors *m* and different skipping parameters *δ* are presented in Table 1.

As shown in Table 1, when keeping the skipping parameter *δ* constant, for the dimension of input vectors *m* varying from 2 to 13, the SampEn results of BBS post time series decrease at first and increase after a critical threshold. For example, when δ=1, for *m* varying from 2 to 11, the SampEn results of the time series decrease from 1.19 to 0.54; however, for *m* varying from 12 to 13, the SampEn results increase to 0.54. Formally, this phenomenon is known as phase transition. In Table 1, it can also be found that, when *m* is increasing, the SampEn results will decreases along with the range of c(m) and c(m+ 1). Similarly, the value of (c(m)−q)/(c(m+ 1) −q) for a given *q* is smaller than c(m)/c(m+ 1) when c(m) is larger than c(m+ 1). When phase transition appears, the complexity of input vector will increase, which means the unpredictability of time series increase.

**Figure 3:**
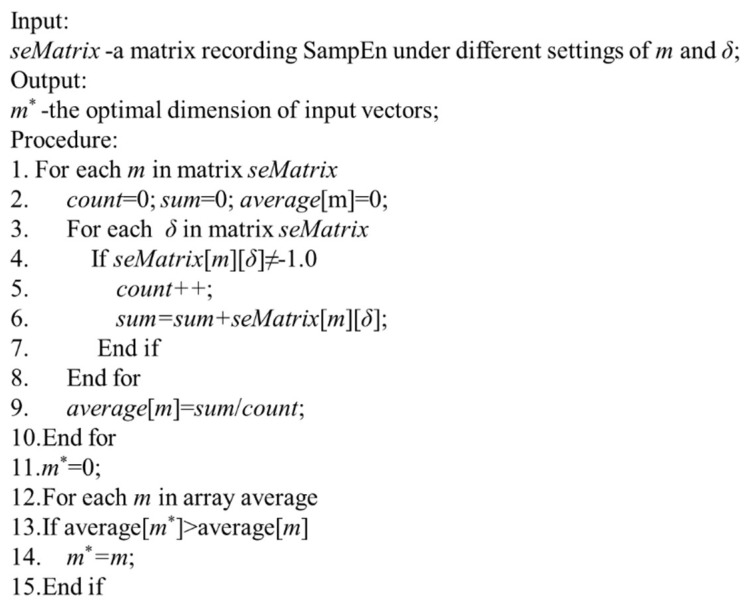
Procedure of determination of the optimal dimension *m*^∗^.

**Figure 4:**
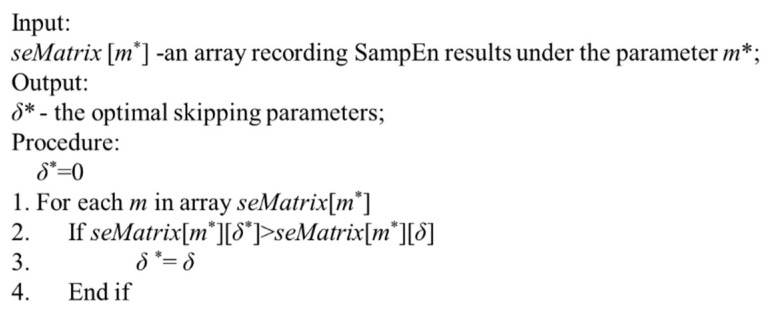
Procedure for determination of the optimal skipping parameter $\delta^{*}$.

Therefore, according to the above analysis, the procedures for the determination of the optimal size of input vector *m* and the optimal skipping parameter *δ* are shown in Figure 3 and Figure 4. At first, for the optimal size of input vectors *m* determination, the average of the SampEn results of different skipping parameter *δ* under same *m* is adopted, and *m* with the smallest average SampEn is selected as the optimal parameter. After that, the skipping parameter *δ* with the minimum SampEn is selected as the optimal skipping parameter.

The procedure for determination of the optimal size of input vector $m^{*}$ is presented in Figure 3. *seMatrix* is derived based on the SampEn results of different *m* and *δ* in Table 1. According to Lines 1–8, the SampEn results of all *δ* under the same size *m* are summarized. Line 9 is to get the average of SampEn results under the same size parameter *m*. Based on the SampEn values in Table 1, the optimal dimension of input vector is obtained as 11. It can be found that, for different dimension of input vector *m*, the possible skipping parameters *δ* with SampEn results not equal to infinity are different from each other.

Therefore, the procedure for determination of the optimal skipping parameter *δ* is shown in Figure 4. According to Lines 2–3, when increasing the skipping parameter *δ* under the dimension of input vector $m^{*}$, the optimal skipping parameter $\delta^{*}$ is found. Based on the SampEn values in Table 1, the optimal skipping parameter *δ* is derived as 5.

## 4. Experiments and Discussions

### 4.1. Datasets

**Figure 5:**
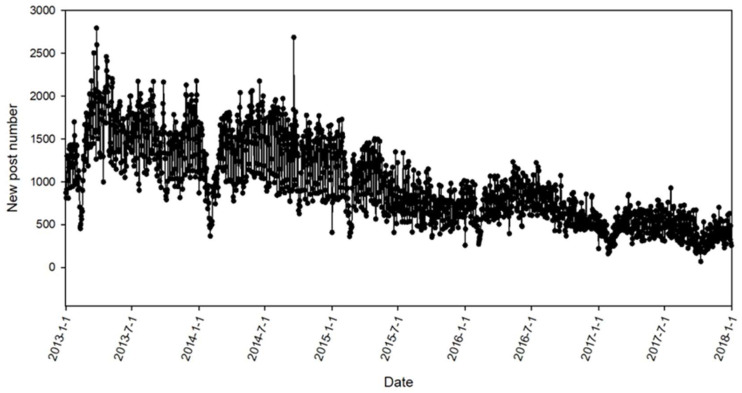
The time series of daily new post number on Tianya Zatan broad.

To compare the effectiveness of SampEn-DNN with the state-of-the-art approaches for time series modeling and forecasting, the time series of daily new post number of Tianya Zantan board (website: http://bbs.tianya.cn/list.jsp?item=free&order=1) was selected as the data source. The daily new posts on Tianya Zatan broad are published by netizens to discuss the hot and sensitive topics of current society. With a spider system developed by our group, the new posts published on Tianya Zatan board were downloaded and parsed, and the numbers of daily new posts were counted. For this study, the numbers of daily new posts from 1 January 2013 to 31 December 2017 were selected. This dataset contains 1826 data points with 1,782,793 new posts. The whole time series of daily new post number is shown in Figure 5.

In Figure 5, it can be found that the time series of daily new post number published on Tianya Zatan shows a downtrend from 2013 to 2017, which is mainly owing to the popularity of Micro-blog and WeChat. The time series of daily new post number also presents periodic fluctuations, e.g., the number of new posts on the working days is much greater than the number on weekends or holidays.

### 4.2. Experimental Procedures

For time series modeling and forecasting, the first issue is to verify whether the time series is stationary. Through ADF test on the BBS new posts time series, it can be found that the dataset is stationary (*P* < 0.05). For effectiveness comparison in daily BBS new post number prediction, the whole dataset was split into five subsets with the daily new posts for each year as one subset (2013, 2014, 2015, 2016 and 2017). Through the ADF test on these five subsets, it can be found that the subset of 2014 is non-stationary. The first order difference was conducted on the five subsets, and then the ADF test results show all subsets are stationary (*P* < 0.05).

ARIMA model: The parameters identification of ARIMA model is based on Box and Jenkin’s approach [27]. After first order differencing manipulation, the time series became stationary. Hence, only the orders *p* and *q* needed to be identified. Traditionally, the charts of autocorrelation faction (ACF) and partial ACF (PACF) [27] are adopted to find several candidate couples *p* and *q*. Furthermore, through the Akaiike information criterion, the two parameters *p* and *q* of ARIMA model were decided. Seasonal ARIMA model: First, considering the influences of seasonal vacations and holidays, the seasonal factor of the time series $S_{t}$ was derived, $S_{t}=7$. Second, with the seasonal factor $S_{t}$, the time series data were filtered through convolution operation. Third, the order of the autoregressive order *p* and the moving average order *q* were also decided based on ACF and PACF charts. Polynomial regression: First, BBS new posts time series was converted to a cumulative time series $N_{new}=\left\{x_{1}^{new},x_{2}^{new},\cdots ,x_{n}^{new}\right\}$, where $x_{i}^{\mathrm{new}}=\sum_{k=0}^{i}x_{k}$. Second, the least squares method was used for parameters identification; Third, the performances of different orders of polynomial regression ewre compared to select the optimal order. ANN model: According to the skipping parameter and input vectors results of SampEn, the training and test dataset were extracted from the time series; then, the reconstructed time series were fed into ANN model to regulate the weights of networks. DNN model: The procedures were similar with ANN model. Based on the results of sample entropy, the training and test datasets were derived, and then used for DNN modeling.

For each subset, the new post number points in the time series of the first 11 months (January–November) were selected as the training dataset, and the new post number points in the time series of the last month (December) were selected as the test dataset. Based on the reconstructed dataset, the predictive performance of SamEn-DNN for BBS post time series was compared with ARIMA, Seasonal ARIMA, polynomial regression, and ANN models. To evaluate the performance of SamEn-DNN and other mentioned methods, magnitude of relative error (MRE) and mean magnitude of relative error (MMRE) were selected as the evaluation criterion. MRE and MMRE are expressed in Equations (18) and (19).

(18) $$MRE_{i}=\frac{\left|x_{i}-{\hat{x}}_{i}\right|}{x_{i}}$$

(19) $$MMRE=\frac{\sum_{i=1}^{n}MRE_{i}}{n}$$

where $x_{i}$ is the real post number and ${\hat{x}}_{i}$ is the estimated post number of model. The smaller are the MRE and MMRE, the better is the prediction. The range of MRE and MMRE are [0,1].

### 4.3. Experimental Results

Based on Equations (18) and (19), the performances of SampEn-DNN and the mentioned methods were evaluated, and the MMRE results are presented in Table 2.

Table 2 shows the MMREs of SampEn-DNN and the mentioned methods for BBS daily new post number prediction. In Table 2, it can be found that, among the five models, SampEn-DNN model shows better performances than other methods on four of the five subsets. Meanwhile, Seasonal ARIMA model shows better performance on one of the five subsets. Thus, it may be concluded that the SampEn-DNN outperforms Seasonal ARIMA model. Furthermore, both SampEn-DNN and Seasonal ARIMA model show better performances on the five subsets than ARIMA, polynomial regression and ANN. Moreover, ANN has produced better performance than ARIMA model, and ARIMA model has outperformed polynomial regression. From this point, it can be said that, among the mentioned five methods, polynomial regression has obtained the poorest performance for BBS new posts time series modeling and forecasting.

To directly show the advantage of each method, the pairwise comparisons of the predictive performances for SampEn-DNN and the other mentioned methods were conducted. The results of MREs of each method on the five datasets were used for comparison. As mentioned in Section 4.2, for each subset, the 31 data points of December were selected as the test dataset, thus there wre totally 155 MREs in the five subsets. For pairwise effectiveness comparison, the Wilcoxon signed rank test [33] was employed. The Wilcoxon signed rank test is a non-parametric statistical hypothesis test for the difference assess of the repeated measurements on a single sample. The Wilcoxon signed rank test results of the MREs of the compared methods are shown in Table 3. The codification of the *P*-value in ranges is defined as follows: “∼” means *P* > 0.05, which indicates that differences in performances of two compared methods are not significant; “<“ (“>“) means 0.01<P≤0.05, which indicates one model slightly outperforms the other model; and “≫” (“≪”) means *P* ≤ 0.01, which indicates one model significantly outperforms the other model. For example, for effectiveness comparison of ARIMA and SampEn-DNN, the code “≪” means that the *P*-value of Wilcoxon signed rank test is < 0.01, thus SampEn-DNN shows a significantly better performance than ARIMA.

From the results in Table 3, it can be found that the outcome of Table 3 validates the conclusions of Table 2. SampEn-DNN has obtained the best performance for time series forecasting of daily BBS new posts, Seasonal ARIMA and ANN are also effective methods for prediction the number of BBS new posts, and Polynomial regression has produced the poorest performance. The results can be explained from two points: (i) SampEn-DNN applies the SampEn method for the dimension optimization of input vectors. Based on the input vectors with smallest complexity, DNN method easily captures the micro-level patterns of BBS new posts time series; (ii) DNN model is a probabilistic, generative model that can learn to probabilistically reconstruct its inputs, and also takes advantage of more neurons to reach the minimum error, thus DNN generates superior performance to the other four methods. In Figure 5, it can also be found that BBS new posts time series show some periodic fluctuations, thus Seasonal ARIMA method shows better performance for this periodically fluctuating dataset than ARIMA. Polynomial regression is in the macro-level and cannot detect the micro-level patterns of post number time series, thus it has produced the poorest performance for BBS new posts time series.

## 5. Concluding Remarks

In this paper, based on SampEn and DNN, a novel approach SampEn-DNN is proposed for BBS new post number time series modeling and forecasting. The multi-scale sample entropy is adopted to optimize the skipping parameter *δ* and the dimension of input vector *m*, and DNN is applied for time series modeling and forecasting based on the optimal parameters. Tianya Zatan board daily new post number was selected as the data source, and extensive experiments based on SampEn-DNN and the state-of-the-art approaches were carried out. From the experimental results, it can be found that, due to the parameter optimization of multi-scale sample entropy, DNN easily learns the micro-level patterns from BBS new posts time series and SampEn-DNN has produced better performance than ARIMA, Seasonal ARIMA, polynomial regression and ANN.

In the future, SampEn-DNN approach will be applied to different tasks on time series modeling and forecasting. For public opinion monitoring, SampEn-DNN will be extended to predict the daily numbers of posts or micro-blogs for more BBSs or micro-blogging websites. Meanwhile, SampEn-DNN approach will be applied to other areas to test its effectiveness, such as weather forecasting, control engineering, etc.

## Figures and Tables

**Table 1:** SampEn (sample entropy) results of BBS (Bulletin Board System) posts time series with different *m* and *δ*.

	*δ*	1	2	3	4	5	6	7	8	9	10	11	12
*m*													
**2**		1.19	1.22	1.20	1.24	1.31	1.32	1.05	1.32	1.36	1.26	1.30	1.38
**3**		1.06	1.06	1.13	1.14	1.09	1.18	0.93	1.20	1.14	1.12	1.18	1.20
**4**		0.96	0.95	1.01	1.05	0.96	1.09	0.85	1.10	1.06	1.04	1.06	1.12
**5**		0.90	0.80	0.91	0.97	0.92	0.95	0.81	1.07	1.00	0.93	0.97	1.15
**6**		0.83	0.75	0.87	0.96	0.82	0.93	0.78	1.00	0.89	0.99	0.94	1.09
**7**		0.81	0.72	0.80	0.90	0.73	0.93	0.76	0.87	0.83	1.13	0.83	0.92
**8**		0.68	0.72	0.90	0.73	0.70	0.85	0.71	0.88	0.97	1.10	0.76	0.78
**9**		0.73	0.72	0.92	0.78	0.61	1.30	0.76	1.14	0.98	0.96	1.10	0.89
**10**		0.63	0.74	0.69	0.98	0.50	1.50	0.74	0.98	1.10	0.92	1.20	1.95
**11**		0.54	0.85	0.59	1.10	0.41	NaN	0.45	NaN	1.11	NaN	NaN	NaN
**12**		0.56	1.50	0.92	NaN	0.47	NaN	0.48	NaN	NaN	NaN	NaN	NaN
**13**		0.98	NaN	NaN	NaN	0.69	NaN	0.62	NaN	NaN	NaN	NaN	NaN

**Table 2:** MMREs (mean magnitude of relative error) of ARIMA (auto-regressive integrated moving average), seasonal ARIMA, polynomial (polynomial regression), ANN (artificial neural networks) and SampEn-DNN (sample entropy-deep neural networks) on BBS post time series.

Subset	ARIMA	Seasonal ARIMA	Polynomial	ANN	SampEn-DNN
1	0.2355 ± 0.0090	0.1968 ± 0.0119	0.2772 ± 0.0109	0.2003 ± 0.0129	0.1419 ± 0.0078
2	0.1895 ± 0.0103	0.1691 ± 0.0126	0.4735 ± 0.0179	0.1694 ± 0.0107	0.1241 ± 0.0078
3	0.1915 ± 0.0117	0.1704 ± 0.0107	0.3535 ± 0.0135	0.1934 ± 0.0092	0.1748 ± 0.0080
4	0.1325 ± 0.0049	0.1188 ± 0.0066	0.3637 ± 0.0212	0.1450 ± 0.0098	0.0878 ± 0.0036
5	0.1653 ± 0.0083	0.1451 ± 0.0102	0.2401 ± 0.0126	0.1494 ± 0.0101	0.1256 ± 0.0043

**Table 3:** Wilcoxon signed rank test on MREs (magnitude of relative error) for the five methods.

Model Pair	Seasonal ARIMA	Polynomial Regression	ANN	SampEn-DNN
ARIMA	<	≫	<	≪
Seasonal ARIMA	∼	≫	∼	<
Polynomial Regression	≪	∼	≪	≪
ANN	∼	≫	∼	<

## References

[1] Guo K., Shi L., Ye W., Li X. A survey of Internet public opinion mining. Proceedings of the 2014 IEEE International Conference on Progress in Informatics & Computing.

[2] Tang X.J. (2013). Exploring on-line societal risk perception for harmonious society measurement. J. Syst. Sci. Syst. Eng..

[3] Pond P. (2016). Twitter time: A temporal analysis of tweet streams during televised political debate. TVNM.

[4] Boecking B., Hall M., Schneider J. (2015). Event prediction with learning algorithms—A study of events surrounding the Egyptian Revolution of 2011 on the basis of micro blog data. Policy Internet.

[5] Wang Y.J. (2017). Stock market forecasting with financial micro-blog based on sentiment and time series analysis. Shanghai Jiaotong Univ. Sci..

[6] Chen X.G., Duan S., Wang L. (2017). Research on trend prediction and evaluation of network public opinion. Concurr. Comp. Pract. Exp..

[7] Yin F., Zhang B., Su G., Zhang R. Research on the public opinion pre-warning based on the logistic model. In Proceeding of the 8th IEEE International Conference on Software Engineering and Service Science (ICSESS).

[8] Zhang J.L. (2015). A study on the evaluation of the analytic hierarchy process based Enterprise network public opinion crisis response. J. Quant. Econ..

[9] Hu H., Wen Y., Chua T.S., Li X. (2017). Cost-optimized microblog distribution over geo-distributed data centers: Insights from cross-media analysis. ACM Trans. Intel. Syst. Tech..

[10] Lan Y.X., Zeng R.X. (2013). Research of emergency network public opinion on propagation model and warning phase. J. Intel..

[11] Yang C., Su G.Q., Lan Y.X., He Y.H. (2014). Research on Emergency network public opinion based on the statistical regression model. J. Chin. People’s Armed Police Force Acad..

[12] Wang H., Li Y., Feng Z., Feng L. (2013). ReTweeting analysis and prediction in microblogs: An epidemic inspired approach. China Commun..

[13] Xu T., Xu X., Hu Y., Li X. (2017). An entropy-based approach for evaluating travel time predictability based on vehicle trajectory data. Entropy.

[14] Men B., Long R., Zhang J. (2016). Combined forecasting of streamflow based on cross entropy. Entropy.

[15] Zhang W., Du Y.H., Yoshida T., Wang Q., Li X. (2018). SamEn-SVR: Using sample entropy and support vector regression for bug number prediction. IET Softw..

[16] Zhang Y.J., Ma J.L., Liu J.L., Xiao S.Z. (2015). Psr-svr network public opinion prediction model. Metall. Min. Ind..

[17] Chen J.D., Zhou X.J., Tang X.J. (2018). An empirical feasibility study of societal risk classification toward BBS posts. Syst. Sci. Syst. Eng..

[18] Jin X.L., Cheung C.M.K., Lee M.K.O., Chen H.P. (2009). How to keep members using the information in a computer-supported social network. Comput. Hum. Behav..

[19] Chen J.D., Tang X.J. (2017). Ensemble of multiple kNN classifiers for societal risk classification. Syst. Sci. Syst. Eng..

[20] Qu Y., Wu P.F., Wang X. Online community response to major disaster: A study of Tianya forum in the 2008 Sichuan earthquake. Proceedings of the 42nd Hawaii International Conference on System Sciences (HICSS’09).

[21] Takens F. (1981). Detecting strange attractors in turbulence. Lect. Notes Math..

[22] Sauer T., Yorke J.A., Casdagli M. (1991). Embedology. J. Stat. Phys..

[23] Richman J.S., Moorman J.R. (2000). Physiological time-series analysis using approximate entropy and sample entropy. Am. J. Physiol. Heart Circ. Physiol..

[24] Govindan R.B., Wilson J.D., Eswaran H., Lowery C.L., Preißl H. (2007). Revisiting sample entropy analysis. Phys. A Statist. Mech. Appl..

[25] Kuremoto T., Kimura S., Kobayashi K., Obayashi M. (2014). Time series forecasting using a deep belief network with restricted Boltzmann machines. Neurocomputing.

[26] Shumway R.H., Stoffer D.S. (2011). Time series regression and exploratory data analysis. Time Series Analysis and its Applications: With R Examples.

[27] Box G.E.P., Jenkins G. (1994). Part one-stochastic models and their forecasting. Time Series Analysis: Forecasting and Control.

[28] Hazewinkel M. (2001). Taylor series. Encyclopedia of Mathematics.

[29] Zhang R., Duan Y., Zhao Y., He X. (2018). Temperature compensation of Elasto-Magneto-Electric (EME) sensors in cable force monitoring using BP neural network. Sensors.

[30] Cantrell C.D. (2000). ‘Vector spaces’ in Modern Mathematical Methods for Physicists and Engineers.

[31] Hinton G.E., Osindero S., Teh Y.W. (2006). A fast learning algorithm for deep belief nets. Neural Comput..

[32] Memisevic R., Hinton G.E. (2010). Learning to represent spatial transformations with factored higher-order Boltzmann machines. Neural Comput..

[33] Mann H.B., Whitney R. (1947). On a test of whether one of two random variables is stochastically larger than the other. Ann. Math. Stat..

